# Supplementary material for: Training a high-performance retinal foundation model with half-the-data and 400 times less compute
Source: Nat Commun. 2025 Jul 25;16:6862. doi: 10.1038/s41467-025-62123-z (PMC12297332; doi:10.1038/s41467-025-62123-z)
Supplement: Supplementary file 1 — Supplementary Information [file 41467_2025_62123_MOESM1_ESM.pdf]

Supplementary Material

S1: Overview of pre-training datasets

	RETFound-MEH	DERETFound	RETFound-Green
**Datasets**	Moorfields Diabetic imAge dataSet (MEH-MIDAS)*, Kaggle EyePACS diabetic retinopathy	AIROGS, DDR, ODIR-2019, Kaggle EyePACS diabetic retinopathy	AIROGS (subsample)*, DDR, ODIR-2019
**Notes**	* MEH-MIDAS is not openly available.	/	*53,327 randomly selected images, 46.8% of the dataset.
**Total images**	904,170	150,786	75,000

Supp. Table 1: Overview of the datasets used for pre-training.

Supp. Table 1 gives an overview of the datasets used for pre-training for the three models. RETFound-MEH and DERETFound only overlap regarding the Kaggle EyePACS diabetic retinopathy dataset, which can be found at: https://www.kaggle.com/c/diabetic-retinopathy-detection/data. The MEH-MIDAS dataset is not openly available.

DERETFound and RETFound-Green have substantial overlap, with RETFound-Green's pretraining data being a subset of DERETFound's. In particular, RETFound-Green used one fewer datasets, and only half of the AIROGS dataset.

Overall, RETFound-Green used slightly less than half of the amount of images that DERETFound used, and about 9% of the amount of images that RETFound-MEH used.

S2: Sensitivity analyses for low-dimensional projections

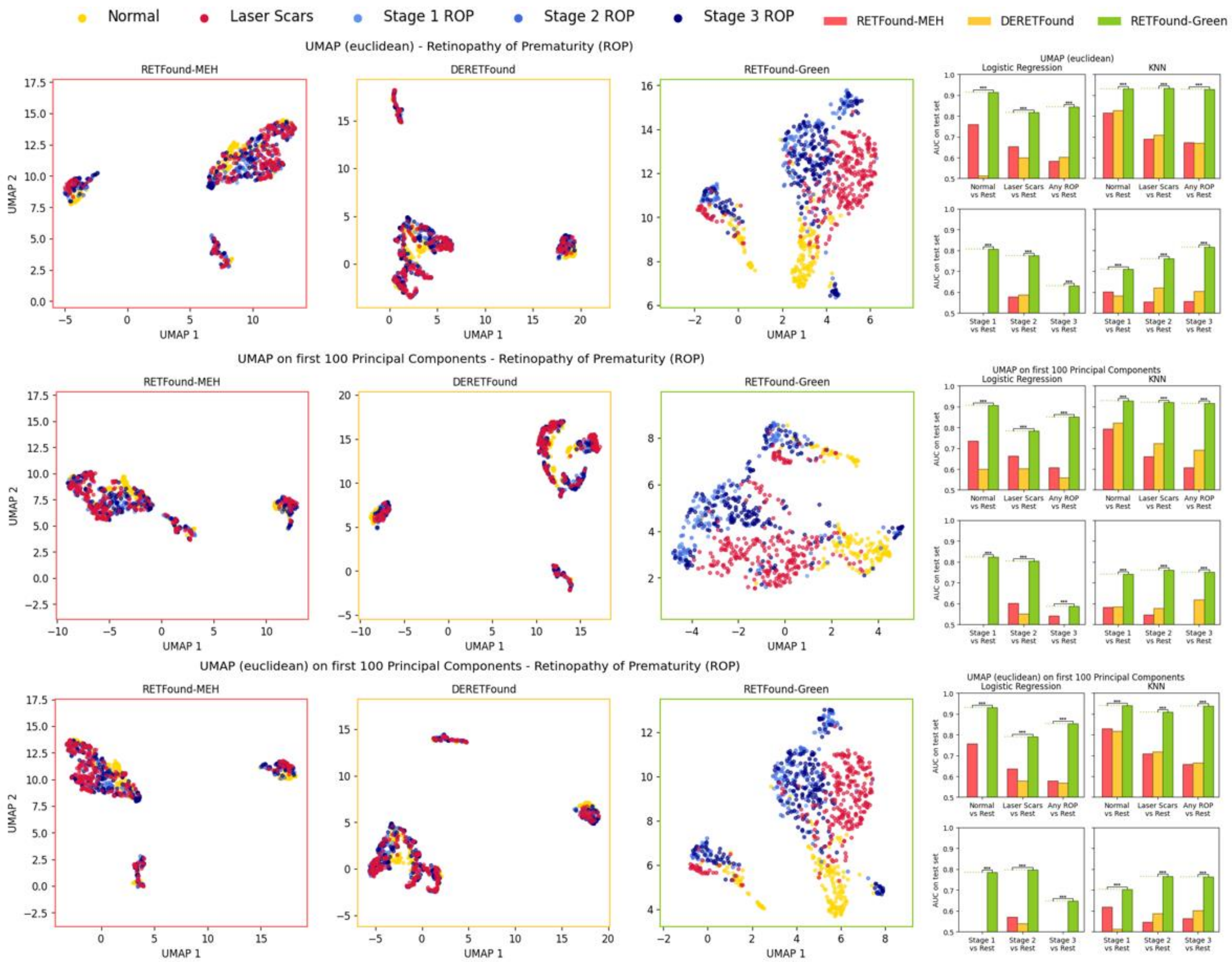

Supp. Figure 1: Two-dimensional projections of the features for the ROP dataset, using (top to bottom): UMAP with Euclidean distance; UMAP on the first 100 Principal Components; UMAP with Euclidean distance on the first 100 Principal Components. Scatter plots show the representations of the train set for each of the three foundation models. The bar plots on the right show the test set AUCs using the two-dimensional projections as input features for different binary targets, using Logistic Regression and KNN as classification algorithms. Missing bars indicate an AUC<0.5, i.e. worse than random guessing. For robustness, reported results are the median of 100 bootstrap samples of the test set. The horizontal bars indicate the result of a Wilcoxon signed-rank test between the best and second best methods across the 100 bootstrap samples, with p<0.05 in bold. “***” indicates p<0.0001.

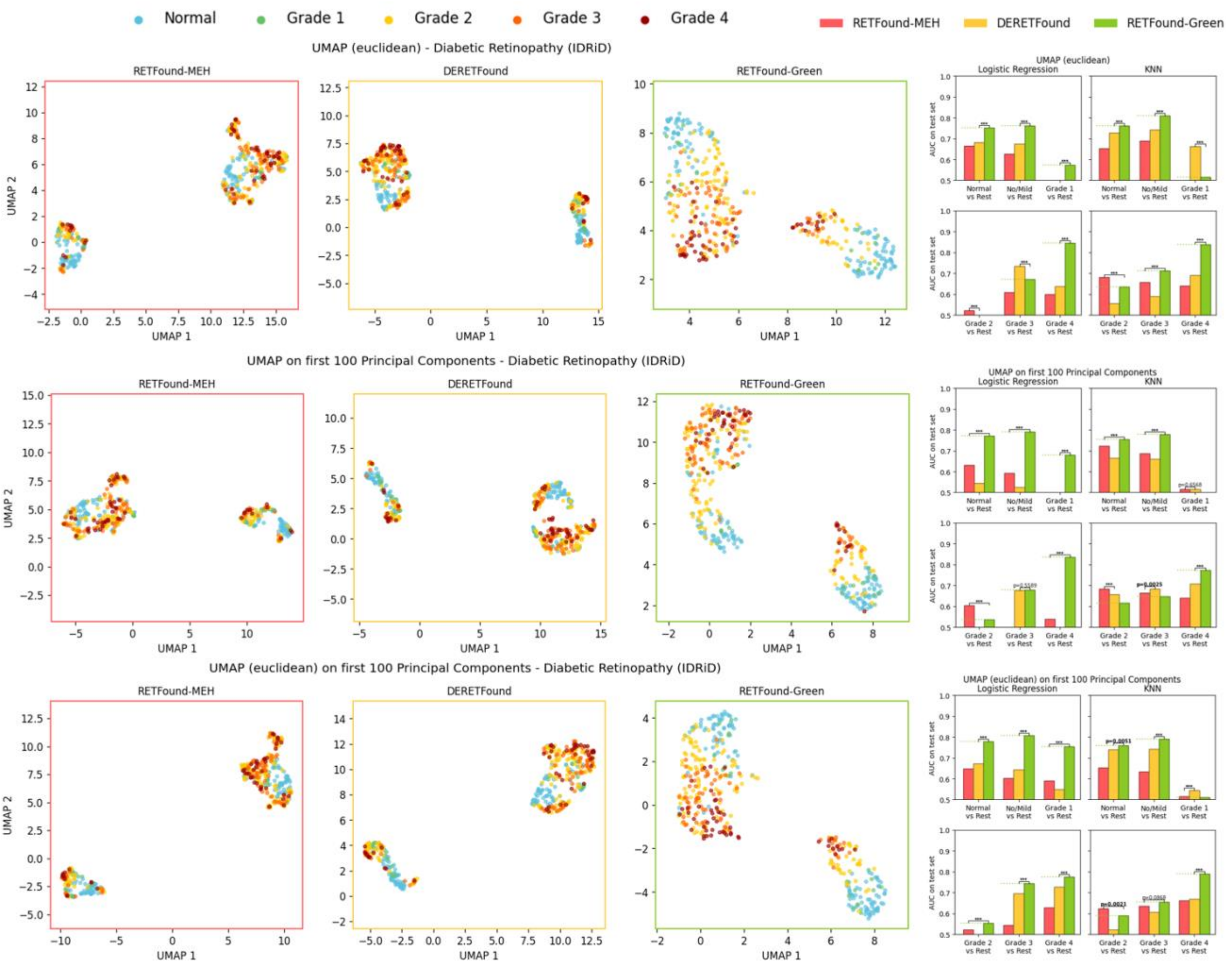

Supp. Figure 2: Two-dimensional projections of the features for the IDRiD dataset, using (top to bottom): UMAP with Euclidean distance; UMAP on the first 100 Principal Components; UMAP with Euclidean distance on the first 100 Principal Components. Scatter plots show the representations of the train set for each of the three foundation models. The bar plots on the right show the test set AUCs using the two-dimensional projections as input features for different binary targets, using Logistic Regression and KNN as classification algorithms. Missing bars indicate an AUC<0.5, i.e. worse than random guessing. For robustness, reported results are the median of 100 bootstrap samples of the test set. The horizontal bars indicate the result of a Wilcoxon signed-rank test between the best and second best methods across the 100 bootstrap samples, with p<0.05 in bold. “***” indicates p<0.0001.

S3: External transportability of low-dimensional projections across BRSET and IDRiD

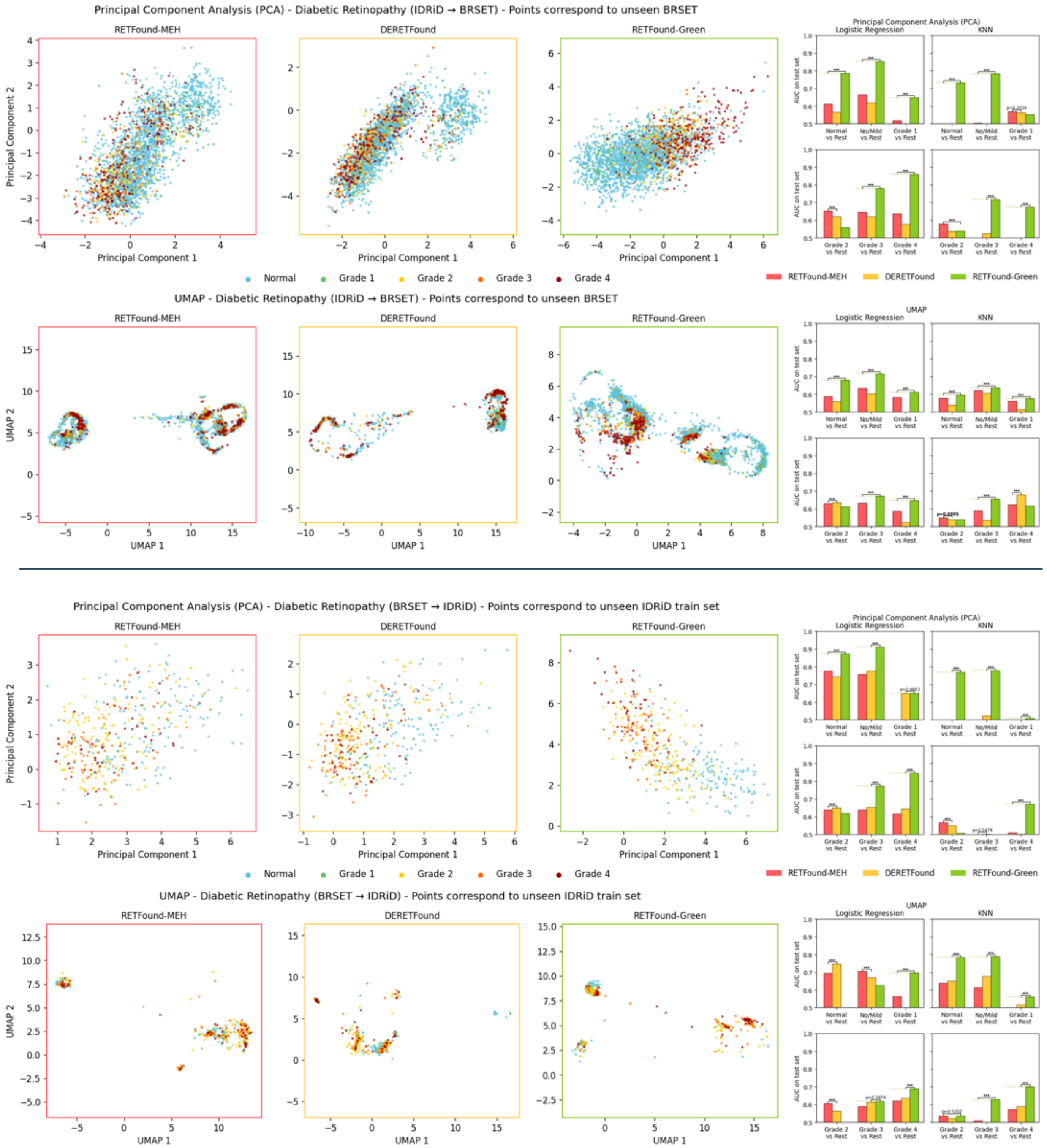

Supp. Figure 3: Two-dimensional projections of features, fitting the projection and classification models to IDRiD and applying them to BRSET (top), and vice versa (bottom). For these experiments, we only include people with diabetes from BRSET which partially alleviates overplotting and reduces the computational burden for UMAP. The bar plots on the right show the test set AUCs using the two-dimensional projections as input features for different binary targets, using Logistic Regression and KNN as classification algorithms. Missing bars indicate an AUC<0.5, i.e. worse than random guessing. For robustness, reported results are the median of 100 bootstrap samples of the test set. The horizontal bars indicate the result of a Wilcoxon signed-rank test between the best and second best methods across the 100 bootstrap samples, with p<0.05 in bold. “***” indicates p<0.0001.

S4: Comparison between RETFound-Green and DinoV2

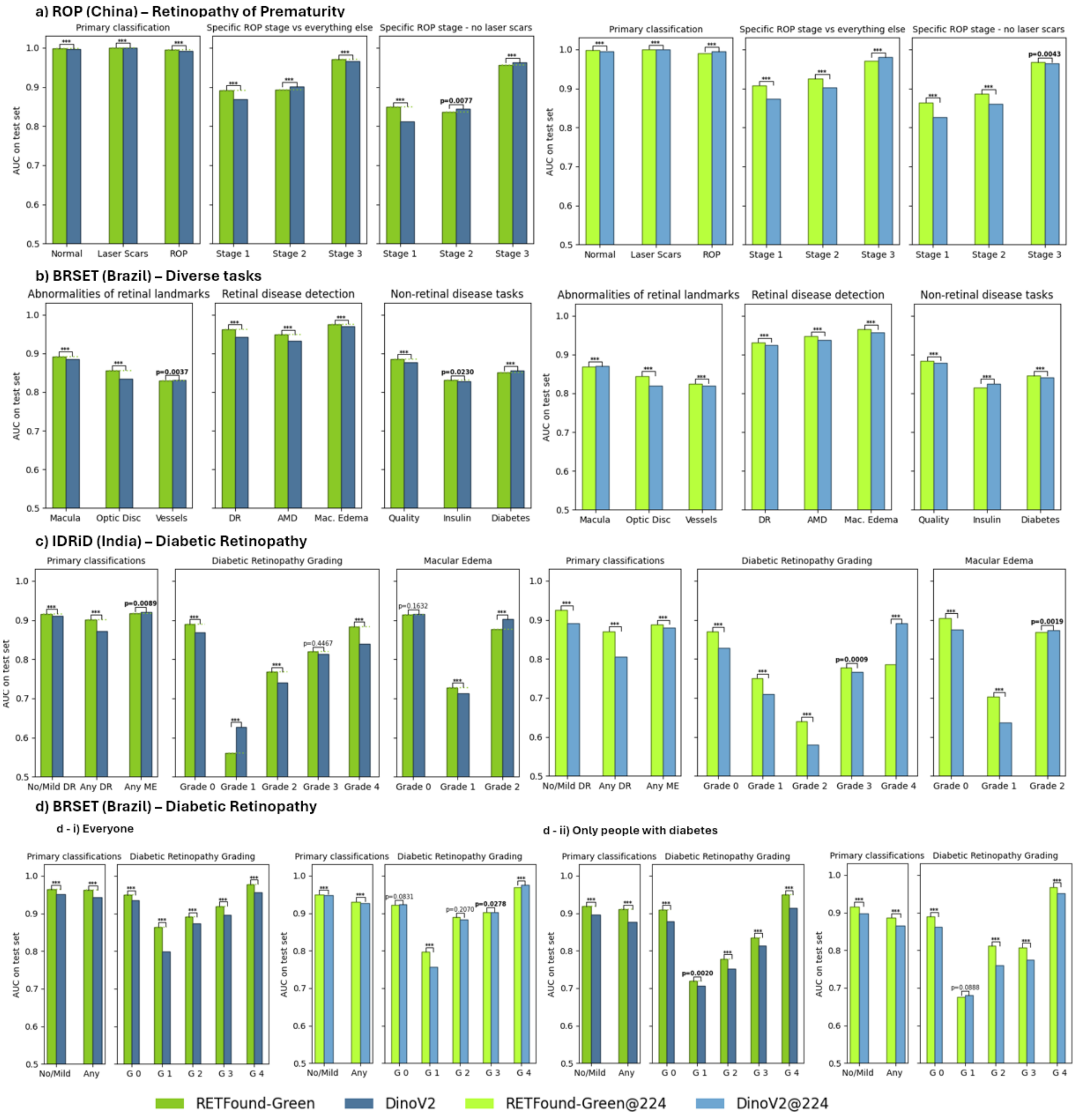

Supp. Figure 4: Comparison between RETFound-Green and DinoV2, at our main resolution of 392x392 and the lower resolution of 224x224. For robustness, reported results are the median of 100 bootstrap samples of the test set. The horizontal bars indicate the result of a Wilcoxon signed-rank test between the best and second best methods across the 100 bootstrap samples, with p<0.05 in bold. "***" indicates p<0.0001.

Supp. Fig. 4 shows the results for the comparison between RETFound-Green and DinoV2, at both 392x392 and 224x224. For ROP dataset, RETFound-Green has 6 wins versus 3 at 392x392, and 7 wins versus 2 at 224x224. For diverse BRSET tasks, it is likewise 7 versus 2 at both resolutions. For IDRiD, at 392x392 RETFound-Green has 6 wins versus 3 and 2 ties, and at 224x224 RETFound-Green has 9 wins vs 2. Finally, for diabetic retinopathy-related tasks in

BRSET, at 392 RETFound-Green wins all 7 comparisons, both when we include everyone and when we only include people with diabetes. At 224x224, RETFound-Green has 3 wins versus 2 and 2 ties when we include everyone. When only people with diabetes are considered, RETFound-Green at 224x224, wins 6 times versus 0 and has 1 tie. Thus, overall, RETFound-Green shows substantially better performance.

S5: Small sensitivity analysis for small downstream adaptation datasets

As a small sensitivity analysis to evaluate whether the relative performance of the three models is vastly different for smaller downstream adaptation datasets, we randomly sample 10 and 20 positive cases for each label. This represents scenarios where we have very limited data, e.g. when trying to develop a classifier for a rare disease. Additionally, we consider the case where all negative cases in the training part of BRSET are available as well as 100 random negative cases being present. These represent the scenarios where we have access to many negatives, e.g. from existing hospital databases, and where we do not have many negatives, e.g. when using a novel type of retinal camera.

We use our default random seed of 42 to select cases. For a fair comparison, the exact same subsets are used for all three models. The same test set as in the main manuscript is used. The results are shown in Supp. Fig. 5. As expected, performance is generally worse than when we have the whole training part of BRSET available for adaptation. However, the results here are consistent with our results in the main part of the manuscript. In fact, RETFound-Green achieves slightly more wins at 10 positive cases per labels than it did with the full dataset. Thus, while these evaluations are not exhaustive, they suggest that our results are robust to changes of adaptation dataset size.

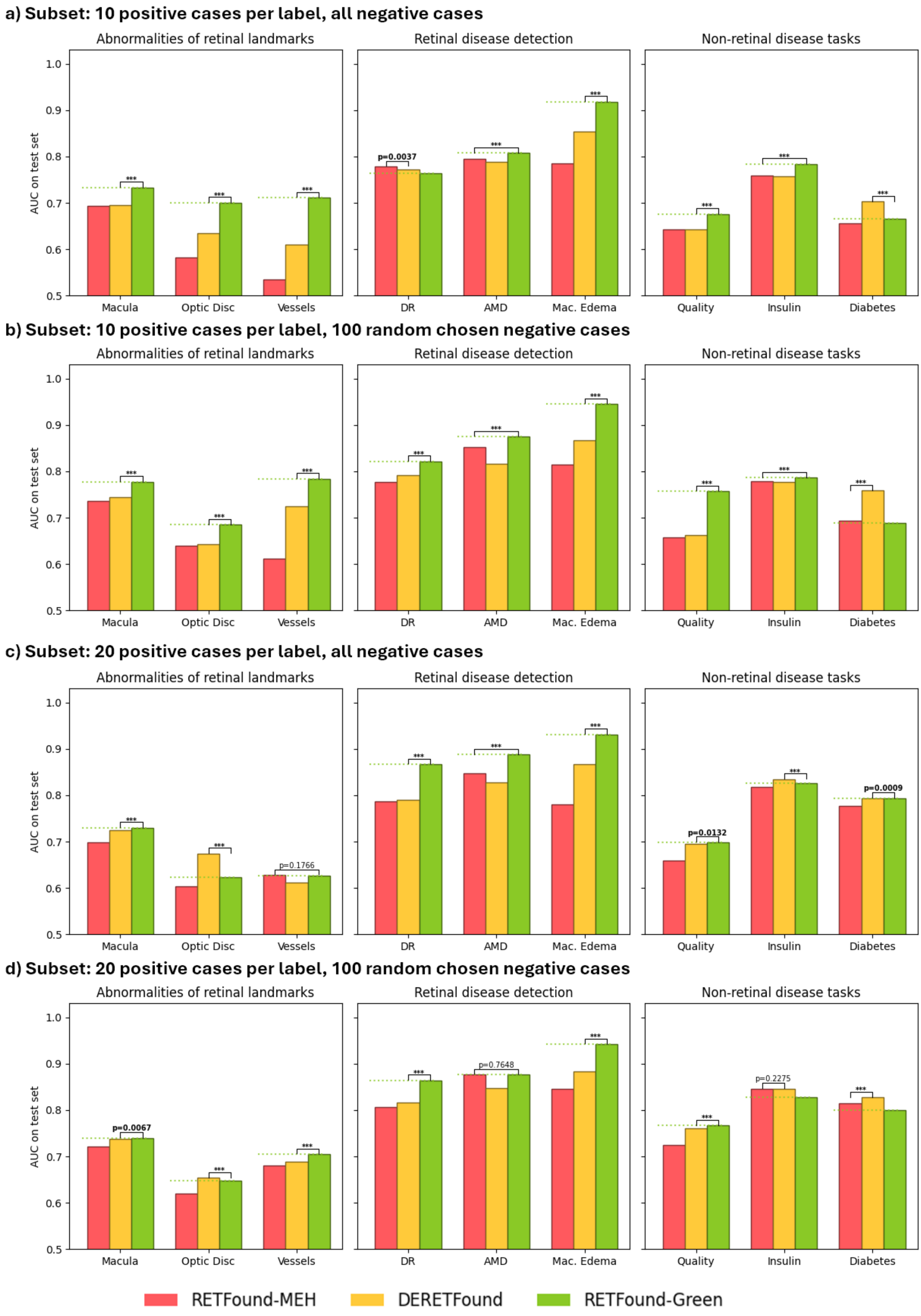
a) Subset: 10 positive cases per label, all negative cases
b) Subset: 10 positive cases per label, 100 random chosen negative cases
c) Subset: 20 positive cases per label, all negative cases
d) Subset: 20 positive cases per label, 100 random chosen negative cases
Abnormalities of retinal landmarks
Retinal disease detection
Non-retinal disease tasks
AUC on test set
1.0
0.9
0.8
0.7
0.6
0.5
Macula
Optic Disc
Vessels
DR
AMD
Mac. Edema
Quality
Insulin
Diabetes
***
p=0.0037
p=0.1766
p=0.0132
p=0.0009
p=0.0067
p=0.7648
p=0.2275
RETFound-MEH
DERETFound
RETFound-Green

Supp. Figure 5: Sensitivity analysis using small subsets of BRSET for training. The horizontal green dashed line indicates the performance of RETFound-Green to aid visual comparison. For robustness, reported results are the median of 100 bootstrap samples of the test set. Best result for each task in bold, the bar with p-value indicates the result of a Wilcoxon signed-rank test between the best and second best methods across the 100 bootstrap samples, with p<0.05 in bold. "***" indicates p<0.0001.

S6: Overview of downstream adaptation datasets

Dataset	Training images	Testing images	Tasks	Data split
**ROP**	866	233	Retinopathy of prematurity related: ROP grading, classifying ROP vs laser scars vs normal	Random 80-20 split at the patient level
**BRSET**	13,013	3,253	Retinal abnormalities for three anatomical landmarks (macula, disc, vessels); Binary retinal disease detection (diabetic retinopathy, age-related macular degeneration, macular edema); non-retinal disease tasks (quality scoring, insulin usage, diabetes mellitus); diabetic retinopathy grading and detection	Random 80-20 split at the patient level
**IDRiD**	413	103	Diabetic retinopathy grading and detection	Official train test split by the dataset creators
**Messidor2**	972	526	Diabetic retinopathy grading and detection	Same data split as in the original RETFound-MEH paper
**Retina**	336	181	Multi-class: Normal vs cataract vs glaucoma vs retinal disease	Same data split as in the original RETFound-MEH paper
**JSIEC1000**	532	318	Multi-class: 39 different classes, we consider 9 classes that are non-trivial as described in the methods section. For more details, please see the original publication by the dataset creators.	Same data split as in the original RETFound-MEH paper

Supp. Table 2: Overview of downstream adaptation datasets.

The data splits for ROP and BRSET will be released alongside our code upon publication. The data splits from the original RETFound-MEH paper are available here: https://github.com/rmaphoh/RETFound_MAE/blob/main/BENCHMARK.md Note that the data splits from the RETFound-MEH paper include a small validation set (also known as "development" or "tuning" set, distinct from the testing set) that we did not use here. However, the validation set was used by the RETFound-MEH authors to select the best fully finetuned checkpoint, which we compare with in this manuscript. Finally, please also note that due to the relatively large size of BRSET, we additionally provide an ablation in Supplementary S6 of using only small parts of BRSET for adaptation of the foundation models.

S7: Results for all classes on JSIEC1000 (including trivial classes)

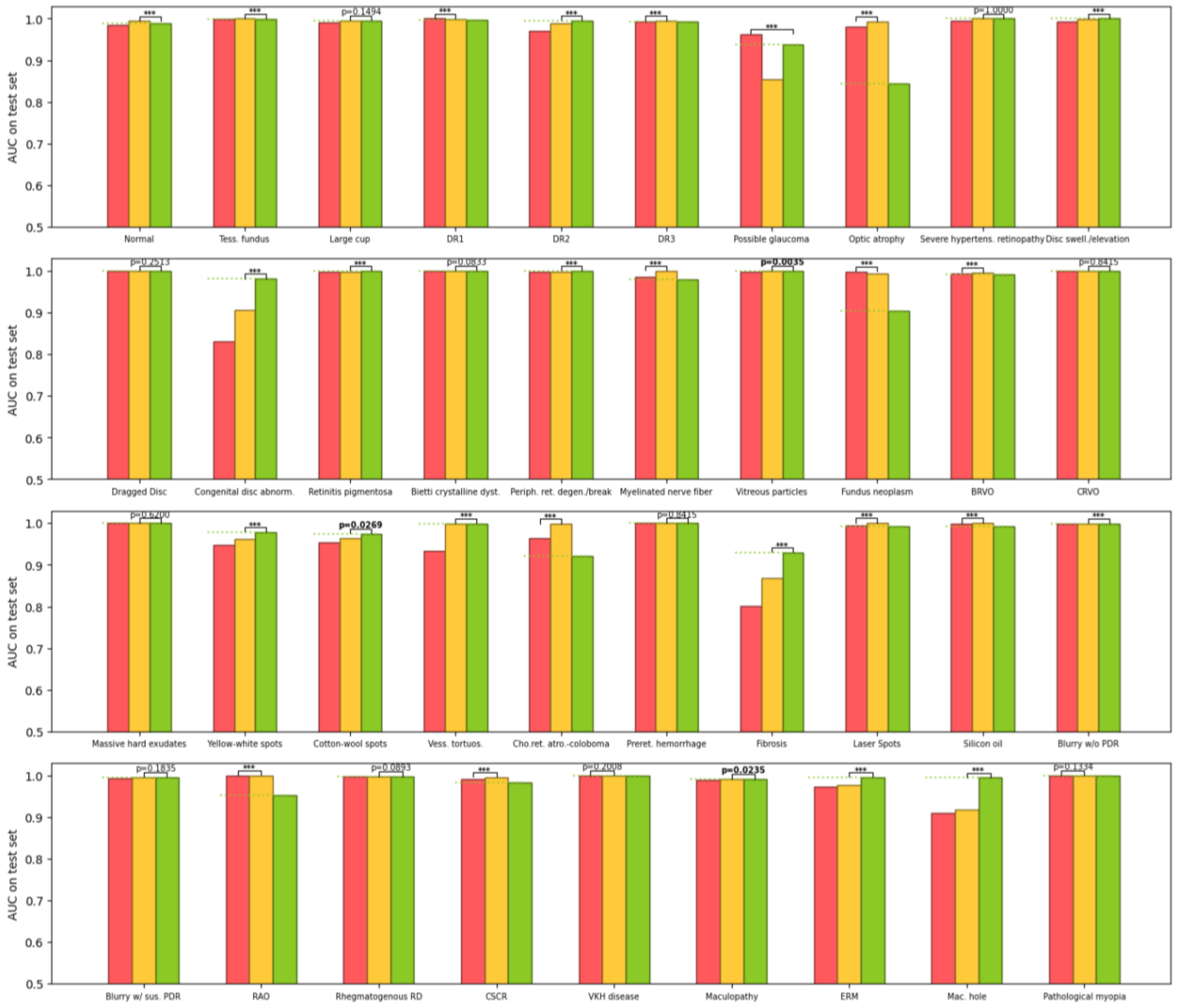

Supp. Figure 6: Full results on the JSIEC1000 dataset, including trivial classes where all models achieve very high performance. The horizontal green dashed line indicates the performance of RETFound-Green to aid visual comparison. For robustness, reported results are the median of 100 bootstrap samples of the test set. Best result for each task in bold, the bar with p-value indicates the result of a Wilcoxon signed-rank test between the best and second best methods across the 100 bootstrap samples, with p<0.05 in bold. "***" indicates p<0.0001.

S8: Effectiveness of the RETFound-Green Token Reconstruction pre-training objective

Our Token Reconstruction objective allows us to train our model in a more efficient way, while yielding a model that is more efficient in downstream usage, too, despite operating at a higher resolution of 392x392 pixels compared to 224x224 pixels used by the other two models. This is a genuine advantage of our approach. However, an interesting question is whether this increase in resolution is the sole reason why RETFound-Green performs so well, despite being far more efficient to train and use. Higher resolutions are known to be beneficial for ImageNet [56]. However, interestingly a substantial part of that improvement is due to higher resolution leading to more total computation per image and thus effectively a higher capacity model, rather than

more information being available at the higher resolution [57]. This was shown by comparing a model at 224x224 and 448x448, as well as using images at 448x448 that were previously downsized to 224x224. Notably, RETFound-Green uses about 2.7 times less computation than RETFound-MEH and DERETFound, while operating at a higher resolution. Thus, RETFound-Green's favourable performance is not due to more computation as a result of a higher resolution.

Nonetheless, we think it is a very interesting question whether our approach would allow for training a good model at the lower resolution. To evaluate the effectiveness of our Token Reconstruction pre-training objective, we thus trained a second model at 224x224 resolution. The results are shown in Fig. 6. "RETFound-Green@224" achieves 7 wins and 1 tie for the ROP dataset, 4 wins for the diverse BRSET tasks, 2 wins and 4 ties for the IDRiD dataset, and 1 win and 3 ties for diabetic retinopathy-related tasks on BRSET for the whole population, and 3 wins and 1 tie when only considering people with diabetes. Thus, RETFound-Green@224 achieves favourable results with at least comparable performance to the other models and the highest number of wins. However, these results are slightly worse than the main RETFound-Green using the 392x392 resolution, indicating that the increase in resolution is beneficial.

Based on the performance of RETFound-Green@224 we can reject the notion that it is solely the increase in resolution that allows RETFound-Green to perform so well, despite using substantially less resources. Additionally, in Supplementary S4, we compare RETFound-Green with the original DinoV2 model at both resolutions. RETFound-Green generally outperforms DinoV2 at both resolutions, further indicating that our Token Reconstruction objective is effective.

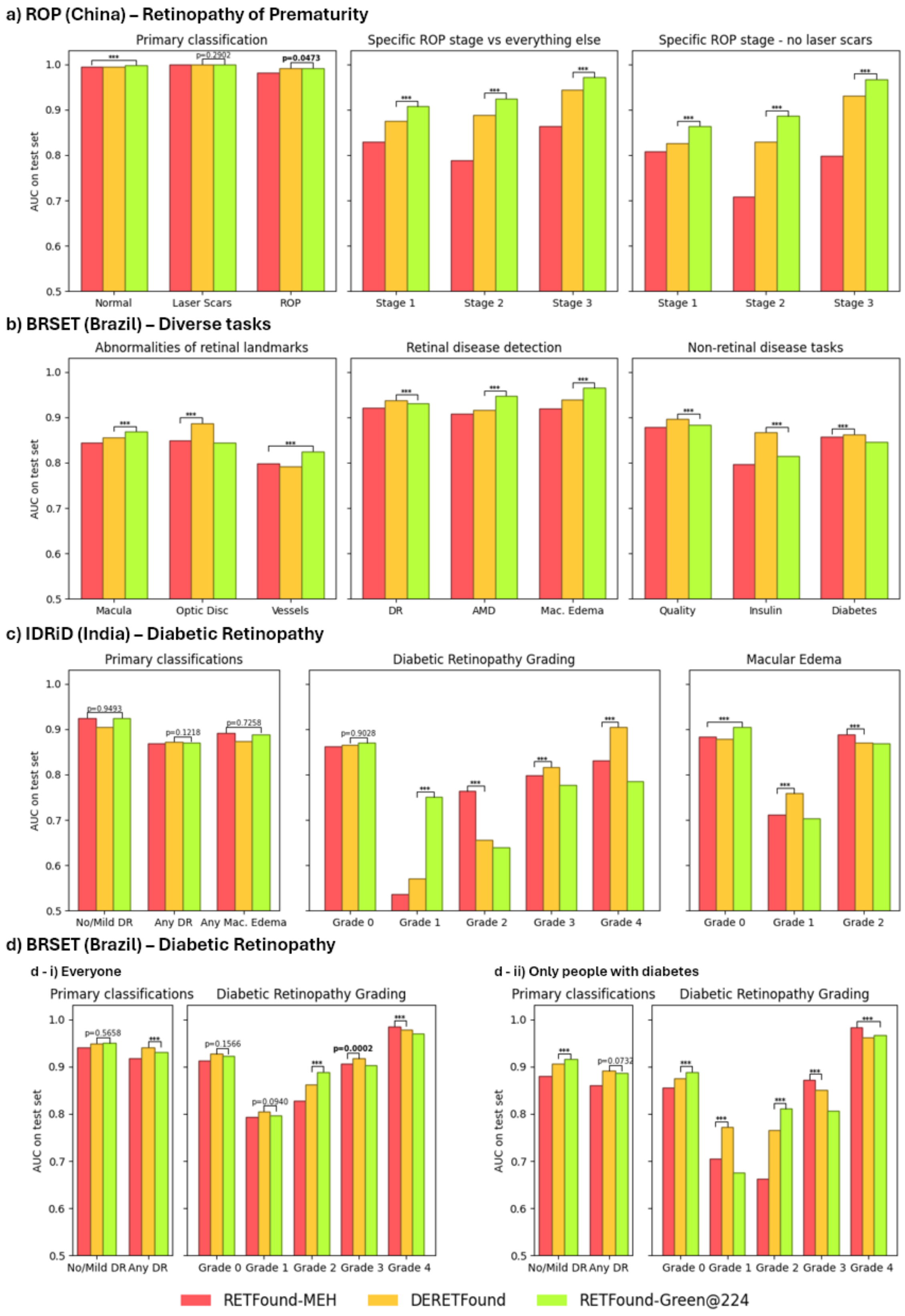

Supp. Figure 7: Performance across a variety of models and tasks, using a RETFound-Green model trained and evaluated at the same 224x224 resolution as the other two models. For robustness, reported results are the median of 100 bootstrap samples of the test set. Best result for each task in bold, the bar with p-value indicates the result of a Wilcoxon signed-rank test between the best and second best methods across the 100 bootstrap samples, with p<0.05 in bold. “***” indicates p<0.0001.

S9: Number of statistically significant wins for each comparison of the three models in the main manuscript

Figure 2		RETFound-MEH	DERETFound	RETFound-Green
ROP - Retinopathy of Prematurity	Wins	0	0	8
	Ties	*0*	*1*	*1*
BRSET - Diverse tasks	Wins	0	4	5
	Ties	*0*	*0*	*0*
IDRiD - Diabetic Retinopathy	Wins	1	2	4
	Ties	*2*	*2*	*4*
BRSET - Diabetic Retinopathy everyone	Wins	1	0	5
	Ties	*0*	*1*	*1*
BRSET - Diabetic Retinopathy diabetes only	Wins	2	1	3
	Ties	*0*	*1*	*1*
Messidor2 - Diabetic Retinopathy	Wins	1	0	4
	Ties	*0*	*2*	*2*
Retina - Multi-class	Wins	0	1	3
	Ties	*0*	*0*	*0*
JSIEC1000 - Multi-class (non-trivial only)	Wins	2	3	4
	Ties	*0*	*0*	*0*
**Total**	Wins	7	11	36
	Ties	*2*	*7*	*9*

Supp. Table 3: Counts of wins and ties for each subplot of Figure 2.

Figures 3 + 4		RETFound-MEH	DERETFound	RETFound-Green
Retinopathy of Prematurity - PCA	Wins	0	0	11
	Ties	*0*	*1*	*1*
Retinopathy of Prematurity - UMAP	Wins	0	0	12
	Ties	*0*	*0*	*0*
IDRiD - PCA	Wins	0	1	10
	Ties	*0*	*1*	*1*
IDRiD - UMAP	Wins	1	1	9
	Ties	*1*	*1*	*0*
**Total**	Wins	1	2	42
	Ties	*1*	*3*	*2*

Supp. Table 4: Counts of wins and ties for each subplot of Figures 3+4.

Figure 5		RETFound-MEH	DERETFound	RETFound-Green
Messidor2 (France) → IDRiD (India)	Wins	1	0	6
	Ties	*0*	*1*	*1*
BRSET (Brazil) diabetes only → Messidor2 (France)	Wins	0	1	6
	Ties	*0*	*0*	*0*
BRSET (Brazil) everyone → Messidor2 (France)	Wins	0	1	6
	Ties	*0*	*0*	*0*

Messidor2 (France) → BRSET (Brazil) diabetes only	Wins	0	2	4
	Ties	*0*	*1*	*1*
Messidor2 (France) → BRSET (Brazil) everyone	Wins	0	3	4
	Ties	*0*	*0*	*0*
IDRiD (India) → Messidor2 (France)	Wins	2	0	5
	Ties	*0*	*0*	*0*
BRSET (Brazil) diabetes only → IDRiD (India)	Wins	1	1	2
	Ties	*2*	*2*	*2*
BRSET (Brazil) everyone → IDRiD (India)	Wins	1	2	3
	Ties	*0*	*1*	*1*
IDRiD (India) → BRSET (Brazil) diabetes only	Wins	1	3	3
	Ties	*0*	*0*	*0*
IDRiD (India) → BRSET (Brazil) everyone	Wins	0	4	2
	Ties	*0*	*0*	*0*
**Total**	Wins	6	17	41
	Ties	*2*	*5*	*5*

Supp. Table 5: Counts of wins and ties for each subplot of Figure 5.

S10: Overview of the datasets used for the external transportability experiments

**Dataset**	**BRSET**	**IDRiD**	**MESSIDOR2**
**Country of origin**	Brazil	India	France
**Continent of origin**	South America	Asia	Europe
**Cameras used**	Nikon NF505 (Nikon, Tokyo, Japan); Canon CR-2 (Canon Inc, Melville, NY, USA)	Kowa VX-10α (Kowa Company, Ltd., Nagoya, Japan)	Topcon TRC NW6 (Topcon Medical Systems, Oakland, USA)
**Diabetes status**	Patients with and patients without diabetes	Only patients with diabetes	Only patients with diabetes

Supp. Table 6: Overview of the datasets used for the external transportability experiments.
